# Impact of kidney function calculation formulae on predicting early adverse renal events in cardiac surgery

**DOI:** 10.1186/cc13560

**Published:** 2014-03-17

**Authors:** M Sileli, F Ampatzidou, S Tsagkaropoulos, K Diplaris, C Koutsogiannidis, G Drosos

**Affiliations:** 1 General Hospital 'G. Papanikolaou', Thessaloniki, Greece

## Introduction

The Cockcroft-Gault (CG) equation and the four-variable Modification of Diet in Renal Disease (MDRD) formula are the most commonly used methods to provide estimation of kidney function. The newly developed Mayo Clinic quadratic equation (MCQE) is another alternative. The scope of this study is to investigate the prognostic value of the above algorithms as prediction models for development of acute renal injury (AKI) and early renal dialysis (RD) in cardiac surgery patients during the postoperative ICU stay.

## Methods

A retrospective single-centre study of 528 consecutive patients admitted to the ICU, who underwent elective cardiac surgery under extracorporeal circulation from July 2012 to November 2013. Patients undergoing urgent or emergent surgery were excluded prior to the study. Preoperative estimation of renal function was obtained using CG, MDRD and MCQE equations. The predictive capacity of these formulae was tested and compared in relation to AKI and RD incidence by constructing receiver operating characteristic curves for each of the models.

## Results

The mean age of the cohort was 64.7 ± 0.45 years and the mean value of estimated kidney function from MCQE, MRDR and CG formulae was 83.7 ± 1.03 ml/minute/1.73 m^2^, 69.03 ± 0.82 ml/minute/1.73 m^2 ^and 75.3 ± 1.18 ml/minute/1.73 m^2 ^respectively. AKI was identified in 75 (14.2%) patients, whereas early RD was necessary in 16 (3%) patients. All of the three variables showed a good predictive value for estimating AKI and RD after cardiac surgery. The area under the curve values for the early RD group was 0.887, 0.867 and 0.804, respectively and for the AKI group was 0.701, 0.651 and 0.691, respectively.

## Conclusion

On the basis of our findings, all of the above algorithms seem to be accurate in predicting AKI and RD incidence in the early ICU postoperative period after elective cardiac surgery. Nevertheless, the MCQE equation more accurately classified individuals compared with MDRD and CG formulae. Our results extend knowledge from previous studies [1],[2]. Further investigations should be performed to determine whether these costless formulae could be used as an additional validated predictor in this group of patients and hence whether they could be incorporated into clinical practice.
